# Emerging trends in diabetes care practice and policy in The Netherlands: a key informants study

**DOI:** 10.1186/1756-0500-7-693

**Published:** 2014-10-07

**Authors:** Michel Wensing, Jan Koetsenruijter, Anne Rogers, Maria Carmen Portillo, Jan van Lieshout

**Affiliations:** Radboud University Medical Centre, Scientific Institute for Quality in Healthcare, P.O. Box 9101, 6500 HB Nijmegen, The Netherlands; University of Southampton, Southampton, United Kingdom; University of Navarra, Pamplona, Spain

**Keywords:** Diabetes care, Self-management, Health policy, The Netherlands

## Abstract

**Background:**

Effective self-management is viewed as the cornerstone of diabetes care. Many interventions and policies are available to support self-management, but challenges remain regarding reaching specific subgroups and effectively changing lifestyles. Here, our aim was to identify emerging policies and practices regarding diabetes care in The Netherlands.

**Methods:**

Study with a purposeful sample of key informants, covering a range of stakeholders. They were individually interviewed, using a flexible and semi-structured approach. A thematic analysis was done, guided by an international framework, which resulted in 28 themes.

**Results:**

After a decade of investing in diabetes care in The Netherlands, stakeholders seem to have shifted their focus towards a view that effective self-management is expected in most people. The expectation is that individuals’ personal networks, community organizations and emerging information technologies will facilitate this. If support of self-management is required, this has to be provided by local coalitions of health and social care organizations, with involvement of municipalities. Poor reach in specific subgroups of the population, such as economically deprived people, is recognized but has not led to targeted policies.

**Conclusions:**

The role of healthcare providers in supporting patients’ self-management in diabetes care seems to be changing in The Netherlands.

## Background

The prevalence of diabetes is rising worldwide, with highest figures in low- and middle-income countries [1]. Life style interventions can effectively reduce the incidence of diabetes type 2 in high-risk patients [2]. In many countries, a range of interventions and policies are being applied to support self-management in people with diabetes. In The Netherlands, diabetes care is largely provided in primary care, involving physicians, nurses, dieticians, physiotherapists and other professionals. On average, about four different disciplines of healthcare providers were involved in the care of a patient with diabetes [3]. In addition, patients’ relatives and community organizations are viewed as having a role to play in the self-management of people with diabetes.

Since 2005, a number of policy measures have focused on optimizing conditions for diabetes care: a national multidisciplinary guideline has been published to guide optimal clinical management, patient education tools are widely available, targeted reimbursement for diabetes care has been created, and improving diabetes care is a prominent target of quality improvement in primary care. These strategies have been enhanced by a nationally convened coalition of stakeholders, which created and implemented a national action plan to improve diabetes care in the years 2008 to 2013 [4]. Despite these efforts, and similar programs in other parts of the world, there remains room for further improvement in diabetes care [5].

As the nationwide effort to improve diabetes care came to an end in 2013, we wondered what new practices and policies were being considered and emerging in diabetes care in The Netherlands. The macro-economic conditions since the year 2008 have been characterized by increasing uncertainty about household incomes, rising unemployment rates, and policies to lower spending on public services, including healthcare. People with chronic conditions are affected by the consequences of this development in a variety of ways, particularly if they live in economically deprived circumstances [6]. The available social systems of support, including personal social networks and community organizations, may counterbalance these potentially negative developments and provide support to efforts of self-management [7].

Stakeholder analysis has been identified in health policy as a method for assessing the viability of future policy options and identifying appropriate strategies and contexts for implementation [8]. Here, our aim was to identify stakeholders’ views on emerging trends in diabetes care practices and policies in The Netherlands, with a particular interest in self-management of people with diabetes.

## Methods

A study involving interviews with key informants was undertaken, following the guidance provided by a written study protocol in the context of the EU-WISE project (available on request from the authors). In The Netherlands, new policies and practices typically first emerge in the health policy arena or in local projects, rather than in the government or related bodies. Therefore we used qualitative research methods to identify what had not yet been documented in national policy papers or formalized programs. The RATS guideline for qualitative research was consulted when writing the paper [9]. This guideline describes a number of features for high-quality publications of qualitative research, which can also be used to design good qualitative studies. All data were handled confidently and reported anonymously. Ethical approval was not required as we did not involve patients in the study.

The study was based on a purposeful sample of 15 key informants, who represented various stakeholders in diabetes care in The Netherlands. Using professional networks, we identified people who could provide relevant information, given their skills or position in society [10]. We planned to include individuals from different stakeholder organizations, covering a range of relevant disciplinary backgrounds and professional roles. Each of these individuals was expected to have knowledge, which was not held by others, thus we were not necessarily expecting saturation in the interviews. The number of 15 informants was pragmatically determined, assuming that a diversity of views could be documented in this way. The participants were interviewed between December 2012 and April 2013 by one of two interviewers, both non-clinician health researchers. The order of the interviews with key informants was pragmatically determined, based on their availability for interviews.

The interviews were designed to identify emerging practices and policies in diabetes care, with a special focus on self-management. The interviews used a few open questions to trigger responses, followed by questions to clarify or illuminate specific items [11]. The interviews started with a broad question (“what are in you view most relevant developments in diabetes care?”), followed by mentioning a few trends relevant for self-management to elicit responses (these items changed over time as a result of interim-analyses), and ending with an open question focused on self-management in people in deprived conditions (“Have macro-economic trends influenced the role of deprivation and thinking about self-management?”). The interviews were undertaken face-to-face or by telephone with the informants at their work setting, and took 30 to 45 minutes each. Directly after each interview, the interviewer made a written summary (paraphrasing closely the responses and including crucial citations ad verbatim) and a short reflection on the content from the perspective of the interviewers. Four interviews were fully recorded and transcribed verbatim to check the adequacy and comprehensiveness of our summaries, which proved to be good. Some respondents provided suggestions for policy documents, from which key points were included in the summaries of the interview.

The qualitative analysis was conducted concurrently with the data collection, led by the first author and involving the second author (both had done the interviews) [11]. An initial analysis after five interviews identified a number of emergent themes. These were elaborated in a further round of interviews, which also included prompts to elicit new themes. This process was repeated after 10 interviews. After all interviews had been completed, we used a modified framework approach to analyze the data [12]. After familiarization with the data, an initial thematic framework was developed inductively from the interview data in The Netherlands. This was followed by a comparison with the frameworks based on similar key informant interviews in five other countries (Bulgaria, Greece, Norway, Spain, United Kingdom), which were done as part of the EU-WISE project. In a two-day international workshop in the Spring of 2013, we developed an integrated thematic framework. The categories of this integrated framework were then applied deductively to the interview data. In an iterative process, further country-specific themes were then identified inductively for each of the categories in this framework. Finally, the interview data were organized according to the identified themes and used to generate interpretations that are provided below.

## Results

Table 1 lists the characteristics of the 15 key informants. We managed to include all stakeholders as planned, but could not reach representatives of relevant industry (e.g. food industry, software developers who were implicated as being relevant respondents as data collection progressed). While we mainly included high-level stakeholders, many of the informants were also active at local level (for instance, as healthcare provider). Table 2 presents an overview of the 28 themes that were identified in the stakeholder interviews in The Netherlands. Quotes are presented to illustrate key points.

**Table 1:** **Descriptive information on the key informants**

	Function	Association	Public sector			Private sector	
			Policymakers	Academics	Managers health units	Non-profit	For-profit
1	Scientific research coordinator	Diabetes charity				**x**	
2	Project coordinator	Health insurer					**x**
3	Policy advisor curative healthcare	Ministry of Health	**x**				
4	Professor in applied health research	University Medical Centre		**x**			
5	Chairman Primary care cooperation/GP/Scientific researcher	Primary care cooperation/GP-practice/University Medical Centre		**x**	**x**		
6	Coordinator diabetes care/GP-expert in diabetes care	Primary care group			**x**		
7	Dietician	Dutch Dietician Organisation/National Programme for Diabetes				**x**	
8	Health consultant/Science practitioner	Local authority/University Medical Centre	**x**		**x**		
9	Staff member ethnic minorities	Senior wellbeing organisation				**x**	
10	Manager	Homecare foundation			**x**		
11	Team leader Prevention and Patient Education/Policy advisor	Dutch College of GPs	**x**			**x**	
12	Quality manager	Dutch Organization of Pharmacists			**x**		**x**
13	Senior policy advisor	Ministry of Health	**x**				
14	Scientific researcher on lifestyle interventions	University Medical Centre		**x**			
15	Consultant community services	Local authority	**x**

**Table 2:** **Themes which were identified in the key informant interviews**

Themes in the international framework	Themes that are specific for The Netherlands
Macro level policies	National government delegates tasks to municipalities
	Municipalities delegate tasks to local organizations
	Municipalities delegate tasks to the individual and his/her network
	Integration of healthcare, social care and prevention
	Diabetes is regarded best practice example for chronic illness care
Recent changes in practices and local policies	Introduction of practice nurses in primary care
	New reimbursement system for diabetes care
	Policies for supporting local communities
	Emergence of online patient education and counseling tools
	Prevention of diabetes remains important
Rationale for changes in policy and practices	Promotion of ‘chronic care model’ to decision makers
	Self-management of disease as way to improve quality of life
	Containment of rising costs of health and social care
	Health information technology as emerging market
Evidence on the impact of changes in practice	Changes in biomedical indicators of diabetes care quality
	Poor reach of in specific subgroups
Factors influencing change of practice	Changes in primary care populations
	Instruments for tailoring to individuals
	Together self-management
	Healthcare providers’ skills
Contextual factors	Financial incentives for primary care
	Price of medication
	Information technology
	Deprivation not on national agenda
Role of stakeholders	Effective lobby by collaboration of stakeholder in diabetes care
	Health insurers
	Primary care
	Municipalities

### National policies

The first category in the framework concerned the national policies regarding health and social care, which are relevant for people with diabetes. In The Netherlands, these reflect a shift of responsibility for tasks at different levels:

#### National government delegation of tasks to municipalities

In recent years, an important principle of the national government has been that many activities are delegated to community level, involving municipalities, because this is expected to help tailoring to local and individual needs. The idea is that self-management support for chronic conditions achieves better integration with other types of support for vulnerable people, such as solving financial debts and housing problems. The focus of the government’s health policy is to organize healthcare close to patients domestic arrangements, in their municipalities.

#### Municipalities delegates tasks to local organizations

Not only the national government is delegating tasks to municipalities, the latter are delegating tasks downwards to local organizations. The role of local authorities is to coordinate and facilitate, but they do not have the financial resources to do much of the practical work. The role of the local authority appears to have shifted from top-down interventions towards more of “a think along” strategy without direct interventions. The shift is towards creating links between all relevant organisations and facilitating local activities. This means that local wellbeing organizations, sport clubs and other organisations are important partners offering possibilities for a more active lifestyle.

“*As local authority we have to withdraw ourselves, (as we are) facing the recent cuts. But we try to place responsibilities as much as possible to those organizations. We encourage and do tell them what we want, and which direction we would like to go.*” [Informant 15]

#### Municipalities delegate tasks to the individual and his/her network

The idea of recent policies of municipalities is as follows. Self management is construed as people being more responsible themselves for their own wellbeing, help by professionals is no longer guaranteed. Instead, the focus of support is seen as emanating from within someone’s own social network. If people need support, then local authorities will check if they can arrange this within their own local connections. Family, neighbours or volunteers can even help to organize this. If this is not possible, the local authorities can still step in. The new policy relies on the idea of self responsibility and less on professional care. This means that a patients’ social network will be used to deliver a structural part of the necessary care and professionals will operate more as coaches to organise and facilitate care. More attention will be given to the support of informal carers so they can deliver more and better care. Only if patients cannot organise the necessary care within their own network will professional help be provided.

#### Integration of healthcare, social care and prevention

The increased role of local organizations and patient networks alongside health professionals creates the need for more cooperation to deliver the necessary care for patients with a chronic condition. To undertake this task, they will have to work together with primary care physicians and other primary care professionals. This collaboration between local organizations and primary care is going to be one of the most important changes in the future care for chronic diseases. An important development is the increased cooperation between GPs, local authorities and community services. Local authorities become more important in the distribution of resources and use these to promote specific activities. Primary care providers know which interventions patients need and can refer them to relevant community services. Therefore it is necessary that GPs become more aware of the possibilities for activities within a local community. Coordination of services is high on the agenda and expected to contribute to reducing the costs of public services. A development is the introduction of the trajectory ‘paradigm’ which intends to reduce fragmentation in health and social care. Each patient is supposed to get his or her own case manager to coordinate the care.

“*That’s what we would like to achieve, to create a vital neighbourhood where the care is efficient. Where professionals thus know which care is offered and can cooperate*” [Informant 15].

### Diabetes is regarded as a best practice example for generic chronic illness care

Diabetes is regarded an “ideal case” for general policies for chronic illness care. There are many diabetes patients, thus there is much attention in the policy world for diabetes, and a set of procedures that can be implemented to improve outcomes for diabetes patients. A previous Minister of Health has invested in care standards and related activities, partly inspired by a study trip to Kaiser Permanente in the US. At the Ministry, the perception is that diabetes is an important topic for primary care physicians, and that they appreciate the information available to illustrate quality of diabetes care, and The Netherlands performs well as compared with other countries.

### Recent changes in practices and local policies

Several changes in practice and local policies were mentioned by the key informants, which were perceived to have impact on diabetes care. Note that many of these changes were coordinated at national level as presented below.

#### Introduction of practice nurses in primary care

An important shift was the transformation of diabetes care from a specialist service (provided in hospitals) to primary care, involving nurses to deliver most of the care. This shift occurred about a decade ago in The Netherlands.

#### New reimbursement system for diabetes care

Additional reimbursement has been created for diabetes care in recent years. In the current situation, the organisation of budgets for diabetes care are stimulating incentives to detect diabetes and to treat it. The knowledge generated by local improvement projects was used to develop criteria for contracting healthcare providers. In addition, the optional additional insurance package (which everyone can choose individually) includes a number of self-management items: a course to stop smoking, membership of a patient organization, participation in physical exercise programs. Some items, such as stop smoking programs, may be included in the basic insurance in the future. It is also increasingly an issue for collective insurance packages (arranged through employers or otherwise, e.g. the elderly association is also a collective). These tend to place value on preventive services more generally.

#### Emergence of online patient education and counselling tools

Another important development in self management is related to e-health. Different organizations are working on developing and implementing internet based portals where patients can access health care providers and manage their own illness related information. For instance, the platform ‘http://www.thuisarts.nl’ is an initiative of the Dutch College of General Practitioners. It offers general information as well as the option to create a personal space. Another example is ‘mijnzorgpagina.nl’ which is an initiative from the Dutch Diabetes Association. This is a website especially for patients with diabetes or other chronic diseases and gives patients the possibility to create a personal profile. The site contains a number of programmes that could help a patient, such as putting together a healthy meal, monitoring blood glucose or other parameters, and checking their body weight. Because patients can create their own profile, they can personalize this website and have directly access to relevant information, support and contacts with other patients. In relation to self-management the sites raise questions and monitoring around whether the patient become more active in their own treatment, and independence in testing and regulating blood sugar.

#### Prevention of diabetes remains a key focus

A controlled study on screening for pre-diabetes did not provide evidence for impact, but early detection of diabetes is still viewed as important. For one local authority diabetes care is not identified as a priority, but promotion of healthy life styles is considered very important, especially to prevent overweight and obesity developing in young people.

### Rationale for and influences on changes in policy and practices

The key informants provided a number of reasons, when asked for the rationale for recent changes in policy and practices. Some of these changes are described in more detail:

#### Promotion of ‘chronic care model’ to decision makers

A major development in policies relevant for self-management was the publication of care standards for a range of chronic diseases in the first decade of the years 2000. These have been developed for diabetes, COPD, and vascular risk management. These care standards aim to support healthcare providers and patients in achieving optimal healthcare for their chronic condition. All care standards recommend that each patient has an explicit (written) individual treatment plan, which is relevant for self-management. This is an agreement between patient and professional about treatment goals and planned activities, including life style changes. The idea of the Chronic Care Model proposed by Wagner has led the development in this field. A more structured approach is expected to lead to a better care and reduced costs.

“*The chronic care model has led in The Netherlands to the development of care standards for diseases such as diabetes. Care standards are documents developed and supported by different stakeholders and are important for policies. Part of those standards is the improvement of self management, individual treatment plans and the implementation of those plans.*” [Informant 4]

#### Self-management of illness as a means of improving quality of life

The government ideology is one in which that there is a great willingness in society to help each other and one which wants to promote the philosophy that people can function independently. It wants to discourage the idea that people have a rightto specific services, simply because these exist and there is regulation that indicates that they are entitled to receive those. A policy for people with chronic conditions and elderly people in general focus on participation (in a broad sense) and the ability to self manage. Participation means to them that people participate in the society and have contact with other people in the neighbourhood and local services. Self management is considered to be the ability to participate without (much) intervention from professionals.

Underlying these policies is a neo-liberal political view that people are responsible for their lives. This implies less focus on the government and on collectively organized prevention of disease. Self-management fits in the broader theme of the quality of life of patients, which is a recognized domain of psychological interventions and research for many health funders. Although self-management applies to patients, it was noted by our respondents that the word is only used by professionals and virtually never by patients. Besides this view on self-management, the most important part of self-management is not dealing with illness but health promotion and prevention of diseases.

#### Containment of rising costs of health and social care

Although cost containment may be linked to self-management in policy rhetoric and plans, the logic underlying this was challenged by several informants. For instance, one did not consider a self management approach as a way to save money and whilst motivational dialogues are expected to result in better health outcomes, although experiences suggest that this is not a less intense way of treatment and not cheaper than traditional healthcare.

#### Health information technology as emerging market

The involvement of private parties (IT companies, pharmaceutical industry, etc.) is welcomed by the national government. These organizations tend to ask little support from the government, except for regulation (e.g. obligatory certification of providers). The Ministry of Health sees private parties as an important source of innovation in healthcare and society more broadly, also in the domain of self-management. This view is consistent with broader policies regarding scientific research of the Dutch government, which seeks to link science closely to economic development.

### Evidence on the impact of changes in practice

We asked the informants about evidence on the impact of changes in practice as a result of emerging trends in practice and policy. Two issues emerged from data:

#### Changes in biomedical indicators of diabetes care quality

One informant reported that there is evidence for increased health outcomes, but not for reduction of costs. However, most key informants did not have a clear view on the evidence for impact of changes in practice.

#### Poor reach to specific population subgroups

Several informants expressed concern about the poor reach in specific subgroups in the population, notably in people in socioeconomic deprived conditions. However, the impact of deprivation on ability to self-manage was perceived to be mixed. Self management programs tend to emphasize one’s own responsibility and it is likely that people from lower socio-economic groups find it harder to join groups on their own. Patients need to master the Dutch language, being capable to plan actions and perform them and need the right motivation to do so. The patients that ultimately join a self management intervention are just a small and very selective group from all patients. On the other hand, deprived areas tend to receive more attention and more professional healthcare workers operate in those areas. Some groups, such as specific ethnic minorities, are well organized, so that they can be reached more easily. However, this is not the case for all relevant subgroups. One of the interventions is to introduce lifestyle coaches who go to peoples own place and help them to join sport or exercise groups. This is a specific intervention for lower SES groups.

### Factors influencing change of practice

When asked for factors influencing desired change in practice (“barriers and facilitators”), the key informants mentioned a number of items. Note that health system-related factors will be discussed in the next section.

#### Changes in primary care populations

A relevant trend in the patient population (besides ageing) are that criteria for diagnosing diseases are stretched (so that more people are diagnosed). Another is that many patients are transferred from hospital to primary care (e.g. after treatment for cancer) so that many vulnerable patients are now under treatment in primary care. As a consequence, more vulnerable patients are dependent on primary care.

#### Instruments for tailoring to individuals

Specific instruments can be used to tailor self-management support to the individual patient. Not everybody can directly start self-management. In one chronic care group, a structured screening tool has been developed and is currently implemented and evaluated in primary care. It distinguishes three categories of patients: individuals who only need some information and can then help themselves, individuals who need to develop some skills first, and individuals who continue to need instruction and are not ready for self-management.

#### Together self-management

An informant at a large health insurer noticed that self-management is only effective “when somebody is standing next to the patient”. This can be a relative, case manager, or coordinating professional. This has emphasized for the health insurer’s policies the direction of ‘together self-management’. A researcher notices that as long as the interventions lasts patients do their exercises, but fail to continue those after the interventions finishes.

#### Health providers’ skills

Healthcare professionals can be examined regarding their skills to provide self-management. Evaluations suggest that there is room for improvement. Nurses in primary care need to be qualified to deliver a self management program.

“*Are all primary care nurses ready to develop self-management interventions? I think this will take some time, especially to develop the necessary skills*” [Informant 14].

### Contextual influencing factors

A number of contextual factors were mentioned by respondents, which were perceived to influence change in practice regarding diabetes care:

#### Financial incentives for primary care

Initiatives to improve care for deprived groups are organised by GPs, but these mostly depend on the initiative of individual GPs and do not belong to common healthcare. According to this respondent GPs do not have financial incentives to organise care and wellbeing beyond their boundaries and they cannot be forced to do so.

#### Price of medication

A substantial number of pharmacies did not sign contracts with health insurers. The implication is that patients have to pay cash in the pharmacy, which is problematic and causes aggression in some cases – particularly in deprived areas. Pharmacies with contracts have different price levels (as a consequence of the health insurer’s policy to discriminate between pharmacies). This is difficult to explain to patients, again particularly in deprived areas. Pharmacists feel that the government falls short in explaining the system to the public.

#### Emerging information technology

A growing number of tools aim to facilitate self-management. Some allow professionals to stay in contact with patients, check and monitor medications without face to face contact. Some information technology firms promote the use of portals to share information with patients. However, the introduction of e-health is not yet implemented widely. This is partly due to the fact that not all elderly are able to work with computers/tablets, although an increasing group of patients can use those devices.

#### Deprivation is absent from the national agenda

Reaching lower economic status groups is not an explicit target of the Ministry of Health and there are no specific policies targeted at this item. The tendency at the Ministry is to argue “these problems can be solved with a little bit of creativity”. Financial deprivation was not an explicit item in the government policies, but one informant thinks that it is emerging. It was defined in terms of specifying different target groups and enhancing the social support system at a local level. This was explicitly presented as an interpretation of signals by this informant rather than a clear fact. There has been some research on the link between SES and health care outcomes in the last decades, but recently there is not much interest on this topic. A focus on self-management might increase the existing gap between groups. Although a self-management program will do no harm, groups with a higher SES will benefit more than lower SES groups.

“*It is likely that groups that needed least care are best capable to organize themselves and live healthy are the best in performing self management as well. And other, more vulnerable groups, are probably less capable in self management and thus increasing the difference between groups*”. [informant 4]

### Role of key stakeholders

The key informants gave also specific information on the role of key stakeholders in diabetes care, which is summarized below.

#### Effective lobbying by collaboration of stakeholder in diabetes care

Diabetes charities collaborate with healthcare providers and patients in the national diabetes federation (NDF). This collaborative has coordinated a four-year action program, which ended in 2013. This was mainly focused on implementation of the ”care standards”: a set of organizational guidelines that intend to facilitate the implementation of clinical guidelines as well as patient empowerment. An informant of the health insurer perceived an increased awareness that a collaborative approach (involving several stakeholders) is needed to make progress. A group of stakeholders met to discuss the possibility of creating a national action plan for self-management. National action plans exist for other topics, e.g. prevention, involve all relevant stakeholders, with the Ministry of Health not necessarily in the lead.

#### Health insurers

An important stakeholder are the health insurers. They work together with local authorities and health professionals to deliver tailored (health) care where it is needed most. Another way health insurers are involved is in their effort to shift care for chronic diseases from hospitals to GPs and homecare. Health insurers do not want to decide what is optimal diabetes care but take the published care standard as starting point for their policies. Health care insurers are not involved in the policy making but do have some influence because they can chose to financially support projects that they think are worth full. For example, the support and education for informal carers was strongly supported by a health care insurer.

#### Primary care providers

The agenda on self-management for chronic diseases is mainly influenced by professional health care organizations. The primary care physicians’ organisation is considered the most influential partner in the policy for self-management.

#### Municipalities

As indicated in the section on national policies, a shift is occurring from national funding of long-term health and social care towards funding by local authorities or health insurers.

## Discussion

This study in key informants provided a view on emerging practices and policies in diabetes care in The Netherlands in the year 2013, with a particular focus on self-management of people with diabetes. After a decade of investing in diabetes care in The Netherlands, and five years after the start of the macro-economic crisis, policy makers seem to agree that effective self-management is desirable in people with diabetes. Effective self-management is viewed as the cornerstone of diabetes care. The expectation is that individuals’ personal social networks, community organizations, and emerging information technologies will facilitate this. If professional support of self-management is required, this has to be provided by local coalitions of health and social care organizations, with involvement of municipalities. Poor reach in specific subgroups of the population, including economically deprived people, is recognized but has not led to targeted policies.

Like all countries, The Netherlands is a special case, which has its unique characteristics. Nevertheless, we believe that many themes are also relevant in other countries. For instance, the macro-economic problems and the prevailing neo-liberal ideology have led to stronger emphasis on self-management of chronic diseases all over the world. Also, a lot of people informally supporting other people exist in many countries, although the configurations and underlying views vary and the precise role and contribution made by a network of personal contacts has not been elaborated. Reaching deprived people with chronic diseases in programs to enhance their health and quality of life is a challenge in most countries. Further studies in the EU-WISE project aim to explore the similarities and differences of these practices and policies across a range of European countries.

This study had strengths and limitations. The flexible approach to sampling and interviews helped to identify items that are not yet well documented. The range of key informants added to the richness of the data. The current study lacks views from relevant industries (e.g. food industry, supermarkets, software developers), although we attempted to include key informants from these stakeholders. We could have included other types of key informants, such as practice nurses. Examination of the available transcribed interviews provided confidence that our interview summaries were a valid starting point for the analysis. Also, the internationally integrated thematic framework provided support to the validity. Nevertheless, we cannot fully rule out the possibility that the non-transcribed interviews contain data that has been missed. We did not attempt to identify deviant cases in the analysis, but it is obvious that several practices and policies are potentially conflicting. For instance, the heavy emphasis on information technology solutions conflicts with the wish to reach deprived people with low literacy skills.

The implementation of self-management support in chronic care management is far from completed in most European countries [13]. The translation of the emerging views and practices into nationwide policies is often a slow and haphazard process. The Dutch healthcare system is characterized by distributed decision making, so that that none of the stakeholders can enforce its views on healthcare. In addition, programs for improving healthcare are complex, implying that its components influence each other and may lead to unpredictable outcomes [14]. Therefore it remains to be seen whether, and how quickly, the identified trends will be widely adopted in practice and policy. This will be influenced by contextual factors, such as the persistence of the macro-economic problems and the dominance of the neo-liberal ideology. The findings of this study are best regarded as a set of options, from which decision makers may chose.

## Conclusions

Effective self-management is viewed as the cornerstone of diabetes care. The expectation is that individuals’ social networks, community organizations, and emerging information technologies will facilitate this. If professional support of self-management is required, this has to be provided by local coalitions of health and social care organizations, with involvement of municipalities.
